# Chemotherapy‐induced neuropathies—a growing problem for patients and health care providers

**DOI:** 10.1002/brb3.558

**Published:** 2016-10-26

**Authors:** Marta Banach, Judyta K. Juranek, Aneta L. Zygulska

**Affiliations:** ^1^ Department of NeurologyCollegium MedicumJagiellonian UniversityKrakowPoland; ^2^ Department of MedicineNew York University Langone Medical CenterNew YorkNYUSA; ^3^ Department of PathologyFaculty of Medical ScienceUniversity of Warmia and MazuryOlsztynPoland; ^4^ Department of OncologyUniversity HospitalJagiellonian UniversityKrakowPoland

**Keywords:** chemotherapy‐induced neuropathies, drug neurotoxicity, neuropathy risk factors

## Abstract

**Introduction:**

Chemotherapy‐induced neuropathies are one of the most common side effects of cancer treatment, surpassing bone marrow suppression and kidney dysfunction. Chemotherapy effects on the nervous system vary between different classes of drugs and depend on specific chemical and physical properties of the drug used. The three most neurotoxic classes of anti‐cancer drugs are: platinum‐based drugs, taxanes, and thalidomide and its analogs; other, less neurotoxic but also commonly used drugs are: bortezomib, ixabepilone, and vinca alkaloids.

**Methods:**

Here, in this paper, based on our experience and current knowledge, we provide a short review of the most common, neuropathy‐inducing anti‐cancer drugs, describe the most prevalent neuropathy symptoms produced by each of them, and outline preventive measures and treatment guidelines for cancer patients suffering from neuropathy and for their health care providers.

**Results:**

Patients should be encouraged to report any signs of neuropathic pain, alteration in sensory perception, tingling, numbness, burning, increased hot/cold sensitivity and motor dysfunctions as early as possible. If known neurotoxic chemotherapeutics are used, a neurological examination with electrophysiological evaluation should be implemented early in the course of treatment so, both patients and physicians would be better prepared to cope with possible neurotoxic effects.

**Conclusions:**

The use of neurotoxic chemotherapeutics should be closely monitored and if clinically permitted, that is, if a patient shows signs of cancer regression, drug doses should be reduced or combined with other less neurotoxic anti‐cancer medication. If not counteractive, the use of over the counter antineuropathic supplements such as calcium or magnesium might be encouraged. If physically possible, patients should also be encouraged to exercise regularly and avoid factors that might increase nerve damage such as excessive drinking, smoking, or sitting in a cramped position.

## 1 Introduction

Every year, a large number of patients undergoing cancer treatment experience peripheral nerve dysfunction and symptoms of polyneuropathy. Distorted thermal sensation, pain and nerve damage are the most common and unpleasant side effects of chemotherapy, affecting treatment dosing, patient well‐being, and quality of life and incurring additional costs on health care systems worldwide (Seretny et al., 2014).

Chemotherapy‐induced polyneuropathies are one of the most common side effects of cancer treatment, surpassing bone marrow suppression and kidney dysfunction. The effects of chemotherapy on the nervous system vary between different classes of drugs and depend on specific chemical and physical properties of the drug used. Observed symptoms range from acute, transient thermal sensation to permanent, accumulative changes in peripheral nerves accompanied by chronic pain and irreversible nerve damage. These symptoms affect patient motor ability, often requiring extensive physical therapy and posing a heavy burden for health care providers (Lema, Foley, & Hausheer, 2010).

Chemotherapeutics exerting neurotoxic effects on the peripheral nervous system are often the ones that are also most commonly used, often listed as a standard, routine medication for most common types of cancer (WHO, 2014). Among them, the three most neurotoxic classes of anti‐cancer drugs are: platinum‐based drugs, taxanes, and thalidomide and its analogs; other, less neurotoxic but also commonly used drugs are: bortezomib, ixabepilone, and vinca alkaloids (Table 1).

Here, in this paper, we aim to provide a short review of the most common, neuropathy‐inducing anti‐cancer drugs, describe the most prevalent neuropathy symptoms produced by each of them, and outline preventive measures and treatment guidelines for cancer patients suffering from neuropathy and for their health care providers.

**Table 1:** Summarized characteristics of neurotoxicity‐inducing drugs routinely used in cancer treatment. List of cancers treated is by no means exclusive, rather we point out to the most frequently treated cancers assigned to a given drug or a group of drugs

Drug name	Types of cancer treated	Mechanism of action	Neuropathy incidence	
Platinum‐based drugs	Lung, ovarian bladder, germ cells testicular, colorectal cancer	Cancer cell DNA‐cross‐linking	Very high	70–100%
Taxanes	Breast, ovarian, lung prostate, pancreatic cancer	Cancer cell microtubule formation impairment	High	11–87%
Thalidomide and its analogs	Multiple myeloma	Antiangiogenesis immunomodulation	High	20–60%
Ixabepilone	Breast cancer	Tubulin malformation	High	60–65%
Bortezomib	Multiple myeloma	Proteasome inhibition	Moderate	20–30%
Vinca alkaloids	Lung, brain, bladder testicular cancer	Cancer cell microtubule formation impairment	Moderate	Up to 20%

## 2 Platinum‐Based Drugs

Platinum‐based drugs, sometimes referred as to platins, such as oxaliplatin, cisplatin, and carboplatin belong to a large class of synthetic anti‐cancer drugs whose main antineoplastic action is triggered by DNA‐cross‐linking‐inhibiting cancer cell DNA synthesis and repair (Kelland & Farrell, 2000). Platinum‐based drugs are enlisted on the WHO Model List of Essential Medicines which represents the most important medications in a basic health system (WHO, 2014) and are used in the treatment of a variety of tumors, from lung and ovarian cancers to bladder, germ cell, testicular, and colorectal cancers.

Neurotoxicity is their main dose‐limiting side effect and affects a large number of platin‐treated patients, with neuropathy incidence rate ranging from 70% to 100% of all treated patients (McWhinney, Goldberg, & McLeod, 2009). These numbers make platinum‐based drugs the most neurotoxic anti‐cancer drugs available on market. Among platins, oxaliplatin evokes the most varied and unique neurotoxic effects, making it detrimental among available classes of anti‐cancer drugs.

Platin‐induced neurotoxic effects include dysesthesias, tingling, and burning sensation and neuropathic pain. Using electrophysiological techniques, peripheral nerve changes are diagnosed as mononeuropathies such as carpal tunnel syndrome or mild to severe sensory and sensorimotor polyneuropathies of axonal origin. Studies suggest that the neurotoxic effects are triggered by drug accumulation in the dorsal root ganglia, causing neuronal dysfunction and apoptosis, thus leading to long‐term, often, irreversible changes in the peripheral nervous system (Avan et al., 2015; Park et al., 2015).

In case of oxaliplatin, additional transient post IV infusion neurotoxic effects have also been observed, which have often been described as jaw tightening, eye pain, leg cramps, pseudolaryngospasm, and cold hypersensitivity. These symptoms usually occur 30–60 min post infusion and resolve within a couple of days (Argyriou et al., 2013; Avan et al., 2015). The mechanisms underlying these transient neurotoxic effects remain unclear; however, recent studies indicate that they might be a direct result of structural properties of oxaliplatin. An oxalate, as part of the oxaliplatin chemical compound, released during oxaliplatin metabolism, binds to calcium through chelation, blocking calcium channels and altering neuronal signal transduction (Avan et al., 2015; McWhinney et al., 2009).

## 3 Taxanes

Taxanes are a class of diterpenoids and include: paclitaxel (Taxol), docetaxel (Taxotere), and cabazitaxel (Jevtana) that act on microtubules, effectively preventing cancer cell division (Abal, Andreu, & Barasoain, 2003). Taxanes have been approved for use in a number of different types of cancer including, but not limited to: breast cancer, ovarian cancer, lung cancer, pancreatic cancer, and recently prostate cancer (cabazitaxel). Within that group, paclitaxel received the status of essential medicine on the WHO Model List of Essential Medicines (WHO, 2014). Despite their widespread use in cancer treatment, taxanes have a number of side effects, one of them being chemotherapy‐induced polyneuropathy. Depending on the taxane used, neuropathy incidence ranges from 11% to 87%, the highest rates of which are reported for paclitaxel (Park et al., 2014; Scripture, Figg, & Sparreboom, 2006). Neurological symptoms include paresthesias and dysesthesias of toes, finger numbness, and loss of dexterity (Pace et al., 1997). Using electrophysiological techniques, patients were predominately diagnosed with moderate axonal sensory neuropathy; however, rare cases of sensorimotor polyneuropathy and carpal tunnel syndrome were observed as well (Vahdat et al., 2001). The mechanism of taxane‐induced neurotoxicity remains elusive; however, growing evidence indicates that a disturbance of axonal transport and neuronal oxidative stress might be the leading factors resulting in neuropathic changes in taxane‐treated cancer patients (Shemesh & Spira, 2010).

## 4 Thalidomide and Its Analogs

Thalidomide (sold as Immunoprin, Talidex, Talizer, or Thalomid) and its synthetic analogs such as lenalidomide, pomalidomide, and apremilast belong to the family of antiangiogenic and immunomodulatory drugs used in multiple myeloma treatment (Hideshima et al., 2000).

Neurotoxic effects of thalidomide are predominately observed in lower extremities neuropathies, such as foot and leg dysesthesia, sensory loss, tingling, impaired reflexes, and painful muscle cramps. Using electrophysiological techniques, thalidomide‐induced neurotoxicity is observed as impaired nerve conduction velocity, altered amplitude, and H reflex absence and in the vast majority of patients is diagnosed as sensory or sensorimotor polyneuropathy of primarily axonal origin. It is estimated that thalidomide‐induced polyneuropathy affects from 20% to 60% of all thalidomide‐treated cancer patients (Cavaletti et al., 2004; Clemmensen, Olsen, & Andersen, 1984; Plasmati et al., 2007). The mechanism of thalidomide‐induced neurotoxicity is not completely clear; however, studies suggest that it might be prompted by nuclear factor‐kappaB‐related dysregulation of neurotrophins that are critical for sensory neuron survival (Briani et al., 2004). Furthermore, growing evidence indicates that old age and pre‐existing predisposition to nerve diseases resulting from a polymorphism of genes involved in nerve repair might play a crucial role in developing a thalidomide‐induced polyneuropathy (Johnson et al., 2011).

## 5 Bortezomib

Bortezomib (sold as Velcade or Cytomib) is a proteasome inhibitor used in multiple myeloma and mantle cell lymphoma treatment. It is a very potent drug, often used when other antineoplastic agents fail (Chen, Frezza, Schmitt, Kanwar, & Dou, 2011; Richardson, Hideshima, & Anderson, 2003). Peripheral nerve changes and polyneuropathy symptoms affect 20–30% of all bortezomib‐treated patients and are manifested as neuropathic pain of toes and/or fingers, tingling and burning sensations, and muscle wasting. Electrophysiological studies show a slight decrease in motor and sensory nerve conduction velocity, H‐reflex prolonged latency and sensory potential low amplitude suggestive of either axonal or demyelination neuropathy (Carozzi et al., 2013; Corso et al., 2010; Thawani, Tanji, De Sousa, Weimer, & Brannagan, 2015). The mechanism of bortezomib‐induced polyneuropathy remains elusive. Recent studies show that one plausible explanation of bortezomib neurotoxicity might be related to its antineoplastic activity targeted at mitochondria that might spread beyond cancer cells and affect neurons. Another possible explanation comes from studies of bortezomib action in cancerous cells, suggesting that its neurotoxicity might be a side effect of blocking NF‐kB activation and subsequent inhibition of nerve growth factors required for neuronal survival. Finally, there is a body of evidence suggesting that bortezomib neurotoxicity might be triggered by inflammatory processes concomitant with neoplastic growth (Argyriou, Iconomou, & Kalofonos, 2008; Meregalli, 2015). Surprisingly, studies showed that cancer therapy involving both bortezomib and thalidomide reduced incidence of chemotherapy‐induced neuropathies in cancer patients, suggesting that both substances might cancel each other's neurotoxic activity in the course of treatment (Chaudhry, Cornblath, Polydefkis, Ferguson, & Borrello, 2008).

## 6 Ixabepilone

Ixabepilone (sold as Ixempra) is an analog of epothilone B and belongs to a relatively new class of anti‐cancer epothilone drugs that act as tubulin destabilizers effectively preventing divisions of cancer cells. Ixabepilone is very potent, however, until now it has only been approved in the US, that is, not in Europe; it is used in breast cancer patients not responding to other available chemotherapies (De Luca et al., 2015; Pivot et al., 2008). Evidence obtained from a large‐scale clinical trial showed that 65% of all ixabepilone treated patients, demonstrated symptoms of neuropathy. In most cases, neuropathies were reversible, mild, or moderate and mainly sensory in nature. Reported symptoms included neuropathic pain, dysesthesia, and neuralgia (Vahdat et al., 2012). As with other chemotherapeutics, the mechanisms underlying ixabepilone neurotoxicity remain unclear. Limited available evidence shows that ixabepilone neurotoxicity might be due to increased deposits of the drug in peripheral neurons, triggering mitochondrial dysfunction and increased oxidative stress in affected neurons (Ebenezer et al., 2014).

## 7 Vinca Alkaloids

Vinca alkaloids, such as vinblastine, vinorelbine, vincristine, and vindesine belong to a class of widely used plant‐derived drugs used in the treatment of different types of cancers, including but not limited to lung, brain, bladder, and testicular cancer (O'Marcaigh & Betcher, 1995). Among vinca alkaloids, vinblastine is the most commonly used anti‐cancer drug, listed in the WHO Model List of Essential Medicines (WHO, 2014). Vinca alkaloids bind tubulin; thus, preventing microtubule formation and inhibiting cancer cell division (Jordan, Thrower, & Wilson, 1991). Vinca alkaloids trigger neuropathy in about 20% of cancer patients; typical symptoms include lower limb weakness, sensory impairment, altered gait, and diminished reflexes. Using electrophysical techniques, nerve changes are diagnosed as predominantly moderate to severe sensory or sensorimotor polyneuropathies (Ness et al., 2013). Mechanisms underlying neurotoxicity of vinca alkaloids remain unclear; however, available evidence suggests that genetic polymorphism of genes crucial for microtubule formation might play a role in developing neuropathy in cancer patients (Diouf et al., 2015).

## 8 Diagnostic Techniques

Neurophysiological techniques and nerve biopsies are useful tools for diagnosis and assessment of functional nerve damage (Boruchow & Gibbons, 2013; Themistocleous, Ramirez, Serra, & Bennett, 2014). Skin biopsies have shown 88.4% diagnostic efficacy (Devigili et al., 2008). A standard skin punch biopsy 3 mm in diameter can be taken 10 cm proximal to the lateral malleolus (Lauria et al., 2010). Morphometric examination of epidermal and dermal nerves is very helpful, especially for investigating small nerve fiber abnormalities, inaccessible to neurophysiological tests. Using immunohistochemical or immunofluorescent techniques, unmyelinated fibers innervating the epidermis and large myelinated fibers and autonomic fibers can be observed (Lauria, Lombardi, Camozzi, & Devigili, 2009).

Conventional nerve conduction velocity studies include the examination of large myelinated fibers; however, the main limitation of this method is its inability to detect changes in small fibers (Themistocleous et al., 2014).

Quantitative sensory testing is another noninvasive, standardized technique for psychophysical threshold evaluation of cold and warm sensations—thermal detection, pain thresholds, and stimuli‐response function (Devigili et al., 2008; Themistocleous et al., 2014). Furthermore, this kind of testing can assess the function of thin and unmyelinated nerve fibers (Verberne, Wiggers, Vermeulen, & de Jong, 2013).

Furthermore, there are new diagnostic techniques such as the measurement of nerve fiber density using corneal confocal microscopy and nociceptive evoked potentials (Hoeijmakers, Faber, Lauria, Merkies, & Waxman, 2012). Nerve morphology can be assessed using in vivo corneal confocal microscopy, including corneal nerve fiber density, that is, the number of nerve fibers/mm^2^, corneal nerve branch density—the number of branch points in the main nerves/mm^2^ and corneal fiber length—the total length of nerves mm/mm^2^ (Ferdousi et al., 2015). In patients with chemotherapy‐induced peripheral neuropathy, using in vivo corneal confocal microscopy revealed an increase in corneal nerve fiber length. This observation correlated with nerve regeneration (Ferdousi et al., 2015).

Nociceptive‐evoked potentials (laser‐evoked potentials and contact heat‐evoked potentials) are neurophysiological and noninvasive methods to evaluate the function of small fiber sensory pathways. This diagnostic method contributes to the early detection of peripheral neuropathy. Latency and amplitude nociceptive‐evoked potentials are associated with the density of intraepidermal nerve fibers. Nociceptive evoked potentials with skin biopsy may be used in the diagnosis of painful neuropathy (Casanova‐Molla, Grau‐ Junyent, Morales, & Valls‐Solé, 2011).

Magnetic resonance neurography has also shown promise as a new diagnostic strategy bringing new insights into the pathophysiology of neuropathies. This method enables visualization of axonal and demyelinating lesions and their localizations in the peripheral nervous system (Wessig, Bendszus, Reiners, & Pham, 2011).

## 9 Pharmacogenetic Techniques

Pharmacogenetic techniques are used to identify genes that may be useful in identifying patients at higher risk of developing chemotherapy‐induced peripheral neuropathies. Nowadays, many trials assessing the interaction between gene‐ and chemotherapy‐induced peripheral neuropathy are conducted. Progressive DNA‐adduct accumulation and inhibition of DNA repair pathways (e.g., extracellular signal‐regulated kinase 1/2, c‐Jun N‐terminal kinase/stress‐activated protein kinase, and p38 mitogen‐activated protein kinase) are the main reasons for peripheral nerve and dorsal root ganglia neuron damage during cisplatin and carboplatin therapy. In case of oxaliplatin, polymorphisms of genes affecting the activity of pivotal metal transporters or voltage‐gated sodium channel genes (e.g., organic cation transporters, organic cation/carnitine transporters, and some metal transporters, such as copper transporters and multidrug resistance‐associated proteins) increase oxaliplatin neurotoxicity and treatment response (Avan et al., 2015). A Japanese study of colorectal cancer patients treated with oxaliplatin‐based chemotherapy estimated the pharmacogenetic correlation between neuropathy and polymorphisms of the excision repair cross‐complementation Group 1 (ERCC1) and glutathione‐S‐transferases pi 1 (GSTP1) genes. The study revealed that polymorphisms of ERCC1, C118T, and GSTP1 Ile105Val made patients more susceptible to oxaliplatin‐induced neuropathy (Inada et al., 2010). Patients carrying CYP3A4 defective variants had more severe neuropathy and higher probability of neuropathy‐induced paclitaxel treatment modifications; hence, CYP3A4 defective variants are a genetic marker associated with paclitaxel treatment modifications caused by neuropathy (Apellaniz‐Ruiz et al., 2015).

## 10 Chemotherapy‐Induced Neuropathies—What Can We Do?

As discussed here, a large body of evidence suggests that chemotherapy‐induced neuropathies are highly prevalent among cancer patients, constituting a major problem both for cancer patients and survivors as well as for their health care providers. The important question arises—is it possible to prevent and/or alleviate chemotherapy‐induced neuropathy symptoms without jeopardizing cancer treatment regimens and incurring additional treatment costs? The answer should be affirmative. It is possible to reduce the number of chemotherapy‐induced neuropathies and lower their financial burden on patients and health care provides, but in order to do so efficiently, we would need to revise patient examination and treatment protocols. Revised protocols should reflect our knowledge of chemotherapeutic neurotoxicity and encourage larger involvement of primary care physicians and neurologists in cancer treatment protocols.

The most important studies concerning preventions and treatment of chemotherapy‐induced peripheral neuropathy, which have been conducted so far, are presented in Table 2. Not only working therapies, but also failed treatment of chemotherapy‐induced peripheral neuropathy has been included in the table. Patients should be encouraged to report any signs of neuropathic pain, alteration in sensory perception, tingling, numbness, burning, increased hot/cold sensitivity, and motor dysfunctions (altered gait, difficulties with walking) as early as possible. Additionally, if known neurotoxic chemotherapeutics are used, a neurological examination with electrophysiological evaluation should be implemented early in the course of treatment; so, both patients and physicians would be better prepared to cope with possible neurotoxic effects. The use of neurotoxic chemotherapeutics should be closely monitored and if clinically permitted, that is, if a patient shows signs of cancer regression, drug doses should be reduced or combined with other less neurotoxic anti‐cancer medication. If not counteractive, the use of over the counter antineuropathic supplements such as calcium or magnesium might be encouraged. If physically possible, patients should also be encouraged to exercise regularly and avoid factors that might increase nerve damage such as excessive drinking, smoking, or sitting in a cramped position.

**Table 2:** Randomized controlled trials concerning prevention and treatment of chemotherapy‐induced peripheral neuropathy according to Brami, Bao, and Deng (2016), with modifications

Author (year)no. of patients	Intervention	Chemotherapy regimen	Results (neurotoxicity)
Argyriou et al. (2006)*n *= 14	Alpha‐tocopherol 300 mg bid during + up to 3 months post chth	Cisplatin	Vitamin E 21.4% vs. control 68.5%
Argyriou et al. (2006)*n *= 16	Synthetic DL‐ alpha‐ tocopherol acetate 300 mg bid during + up to 3 months post chth	Paclitaxel	Vitamin E 18.7% vs. control 62.5%
Pace et al. (2010)*n *= 17	Alpha‐tocopherol 400 mg bid before chth (median 3 months) + 3 months after chth	Cisplatin	Vitamin E 5.9% vs. placebo 41.7%
Kottschade et al. (2011)*n *= 96	DL‐alpha‐tocopherol 400 mg bid during + 1 month after chth	Taxanes, cisplatin, carboplatin, oxaliplatin, combinations of taxanes	Vitamin E 34% vs. 29% placebo
Afonseca et al. (2013)*n *= 18	Alpha‐tocopherol 400 mg/day, 5 days before until chth completion	Oxaliplatin	Cumulative incidence: vitamin E 83% vs. placebo 68%
Wang et al. (2007)*n *= 42	Glutamine 15 g/bid for 7 days 2 weekly + usual care starting chth day 1, day 42	Oxaliplatin	Neurotoxicity G1 after 2 cycles: glutamine 16.7% vs. control 38.4%, G3‐4 after 4 cycles: glutamine 4.8% vs. control 18.2%, G3‐4 after 6 cycles: glutamine 11.9% vs. control 31.8%
Loven et al. (2009)*n *= 23	Glutamine 500 mg t.i.d throughout chth to 3 weeks after completion treatment	Paclitaxel + carboplatin	No significant differences, but neurotoxicity presented with lower severity in glutamine group vs. placebo
Nishioka et al. (2007)*n *= 22	Goshajinkigan (GJG) 7.5 g/daily throughout chth	Oxaliplatin	Neurotoxicity G3: GJG 0% vs. control 12%
Kono et al. (2013)*n *= 44	Goshajinkigan 7.5 g/daily for 26 weeks from chth start	Oxaliplatin	The incidence of neurotoxicity G2 or greater after 8 cycles: GJG 39% vs. placebo 51%
Hershman et al. (2013)*n *= 208	Acetyl‐L‐carnitine 3000 mg/daily for 24 weeks	Taxanes	Acetyl‐L‐carnitine significantly increases neurotoxicity by 24 weeks of treatment
Guo et al. (2014)*n *= 122	Alpha lipoic acid 1800 mg/daily for 24 weeks except 2 days prior – 4 days after chth	Platinum derivatives	No differences between groups (alpha lipoic acid vs. placebo)
Ghoreishi et al. (2012)*n *= 35	Omega 3 fatty acids 640 mg t.i.d during chth + 1 month after chth	Paclitaxel	Omega 3 fatty acids lowers neuropathy by 70%
Rostock et al. (2013)	Electroacupuncture, *n *= 14, hydroelectric baths, *n *= 14, vitamin B1 300 mg/daily/B6 300 mg/daily, *n *= 15, placebo, *n *= 17,	Taxanes, alkaloids vinca, platinum derivatives	No differences between groups
Rao et al. (2007)*n *= 50	Pregabalin (target dose 2700 mg daily)	Paclitaxel, docetaxel, cisplatin, carboplatin, oxaliplatin, vincristine, vinblastine	No significant differences between study arm (pregabalin vs. placebo)
Shinde et al. (2016)*n *= 23	Pregabalin 75 mg bid within 12 weeks of chth	Paclitaxel	No significant differences between study arm (pregabalin vs. placebo)
Cascinu et al. (2002)*n *= 21	Glutathione 1500 mg/m^2^ before oxaliplatin	Oxaliplatin	Neurotoxicity G2‐G4 after 12 cycles: three patients in glutathione group vs. eight in placebo group
Milla et al. (2009)*n *= 27	Glutathione 1500 mg/m^2^ before oxaliplatin	Oxaliplatin	Significant reduction of neurotoxicity in glutathione group vs. placebo
Smyth et al. (1997)*n *= 74	Glutathione 3 g/m^2^ before cisplatin	Cisplatin	Neurosensory toxicity: 39% in glutathione group vs. 49% in control group; neuromotor toxicity: 9% in glutathione group vs. 12% in control group
Yang et al. (2012)*n *= 39	Duloxetine 30 mg/daily to 60 mg/daily	Oxaliplatin	63.3% patients had a VAS score improvement and grade of neurotoxicity improvement
Matsuoka et al. (2012)*n *= 15	Duloxetine in maintenance dose 20–40 mg/daily		Pain was reduced in 7 out of 15 patients
Smith et al. (2013)*n *= 220	Duloxetine 30 mg/daily	Taxanes, platinum derivatives	The mean difference in average pain score between duloxetine and placebo was 0.73
Kemp et al. (1996)*n *= 120	Amifostine 910 mg/m^2^ every 3 weeks	Cisplatin	Amifostine reduced neurotoxicity of cisplatin
Lorusso et al. (2003)*n *= 93	Amifostine 910 mg/m^2^ every 3 weeks	Carboplatin + paclitaxel	Neurotoxicity: 3.7% in amifostine group vs. 7.2% in control group
De Vos et al. (2005)*n *= 42	Amifostine 740 mg/m^2^ every 3 weeks	Carboplatin + paclitaxel	Amifostine decreased neurotoxicity 2–3 grade
Openshaw et al. (2004)*n *= 14	Amifostine 740 mg/m^2^ every 3 weeks	High‐dose paclitaxel	Ineffective in preventing/reducing neurotoxicity
Hilpert et al. (2015)*n *= 40	Amifostine 740 mg/m^2^ every 3 weeks	Carboplatin + paclitaxel	No significant differences between groups (amifostine vs. placebo)

Chth, chemotherapy; G, grade; neuropathy stages.

## Conflict of Interest

None declared.
